# Dietary Behaviors and Caregiver Perceptions of Overweight and Obesity among Chinese Preschool Children

**DOI:** 10.3390/ijerph15040716

**Published:** 2018-04-11

**Authors:** Amber Tang, Meimei Ji, Yefu Zhang, Jiaojiao Zou, Mingzhi Li, Lina Yang, Qian Lin

**Affiliations:** 1 Department of Molecular, Cellular, and Developmental Biology, Yale University, 219 Prospect St., New Haven, CT 06511, USA; amber.t30@gmail.com; 2 Department of Nutrition Science and Food Hygiene, Xiangya School of Public Health, Central South University, 110 Xiangya Rd., Changsha 410078, China; jimeimei1024@foxmail.com (M.J.); yefuzhang@foxmail.com (Y.Z.); zjj170605@foxmail.com (J.Z.); lmz1976@126.com (M.L.), ylnly1997@csu.edu.cn (L.Y.)

**Keywords:** pediatric obesity, China, dietary behaviors, caregiver perception

## Abstract

*Introduction*: Early childhood obesity in China has become a pressing public health concern. A substantial barrier to healthy weight management is poor parental recognition of child overweight. This study examined the relationship between caregiver perceptions of child weight and dietary practices. *Methods*: A total of 364 children between 2 and 6 years old from six urban preschools in Changsha (China) were included in a cross-sectional study. Information on household demographics, health behaviors, and caregiver attitudes was collected through a self-administered caregiver questionnaire. Chi-squared tests, *t*-tests, and multivariable logistic regression analysis were used to determine the relationship between caregiver perceptions, dietary behaviors, and child weight status. *Results*: Over 60% of caregivers with overweight/obese children underestimated their children’s weight status. These caregivers were less likely to worry about weight and restrict their children’s dietary intakes. Children of caregivers who incorrectly classified their weights were also more likely to have a poor appetite. Caregivers of male children and those from families with incomes between 7000 and 11,000 Ren Min Bi (RMB) were more likely to underestimate weight compared to caregivers with daughters and those from higher income households. *Conclusions*: Although accurate weight perception may be important for motivating healthy behavioral changes, it may also lead to greater restriction of children’s diets, which has been linked to long-term weight gain. Interventions to improve awareness of child overweight should be coupled with efforts that teach caregivers about healthy weight management strategies.

## 1. Introduction

Since the late 1980s, China has experienced a rapid rise in overweight/obesity, especially among young children. Early childhood obesity has become a major public health problem in China, with an estimated prevalence of 6 to 14% in urban, preschool-aged children [1,2,3,4]. Rapidly increasing burdens of childhood overweight/obesity have been attributed to recent modernization and nutritional transitions [5,6]. Levels of pediatric overweight in China have more than doubled since 1991, reaching an estimated 15% in 2011 [7]. This prevalence is similar to that of Western countries, such as the US, where 15% of children were also reported to be overweight in 2011–2012 [8,9,10]. Obesity during childhood is a strong predictor of obesity in adulthood and has been associated with poor cardiovascular health, in addition to elevated risk for type 2 diabetes, hypertension, and other non-communicable diseases [11,12,13,14]. Dietary habits have also been shown to form during early childhood and persist into adolescence and adulthood [15]. Therefore, early interventions that target young children are crucial [16].

Parents have a large influence on the eating behaviors of their children as they control the energy density and portion sizes of the foods that are consumed [17,18,19]. Parental involvement has been shown to be critical in efforts to reduce childhood overweight/obesity [20]. However, for parents to make positive changes in their children’s diets, they should have an accurate perception of their children’s weight statuses and understand the potential health consequences [21,22]. Studies in Europe and the US have shown that parents frequently underestimate their children’s weights, especially those of overweight/obese children [23,24]. A study conducted in Italy showed that a third off caregivers surveyed believed that greater child weight was an indication of good health. Caregiver education was also positively associated with the identification of childhood overweight as a health concern [25]. In China, where grandparents or other family members are commonly the primary caregivers of children, studies have reported similar results with 40 to 72% of caregivers underestimating the weights of their overweight children [26]. Furthermore, 65% of caregivers in China with an overweight child reported that they would not want to decrease their children’s weights [27,28,29]. Little is known, however, about how caregiver attitudes on weight status relate to health behaviors in young, Chinese children. 

Accordingly, the aim of this study was to understand how dietary behaviors and caregiver perceptions of child weight are related to child weight status among Chinese 2- to 6-year-old children. We conducted an observational, cross-sectional study in six urban preschools in Changsha (China). Findings from this study may be used to inform future interventions on the management of pediatric overweight/obesity among Chinese children.

## 2. Methods

### 2.1. Study Population

This study was a cross-sectional investigation conducted between June and October of 2017 in Changsha, the capital of the Hunan Province in southern China. Random cluster sampling was used to select classes from six urban, public preschools in Changsha. In each school, one class from every grade (lower, middle, and upper) was recruited. All children in the class were invited to participate in the study. In total, 424 children aged 2-to-6-years-old (210 male and 214 female) across all six preschools were enrolled. Written informed consent and verbal assent were obtained from all caregivers and children who participated in the study. Ethical approval was obtained from the Human Subjects Committee at Yale University (ID: 2000020931) and the Xiangya School of Public Health IRB (No. XYGW-2017-22). 

### 2.2. Data Collection

All information collected from caregivers was obtained through a self-administered questionnaire, which was a practical and efficient instrument through which to collect data. Primary caregivers were defined as those who regularly cared for the children, such as mothers, fathers, grandparents, or nannies. Demographic data, including sex, age, birth weight, number of siblings, household income, and caregiver education level, were collected through the primary caregiver questionnaire. Caregivers were also asked about children’s dietary behaviors, such as whether they had a fixed time and location for meals, how much time they spent at meals, if they engaged in other activities while eating, if they held food in their mouths, and if they had a poor appetite. Engaging in other activities while eating, holding food in the mouth, and having a poor appetite may be indicative of feeding difficulties [30]. Dietary intake was assessed using a 38-item food frequency questionnaire (FFQ), which was modified from a previous study and validated in a pilot study. FFQs were previously shown to be a useful tool for collecting data on dietary intake [31]. Caregivers were asked how often a certain food was consumed in the past week and selected from nine possible responses, ranging from “never” to “5–6 times a week” to “≥3 times a day.” Dietary diversity scores (DDS) were calculated by summing the number of food categories (starches; legumes; dairy; meat, poultry, fish, and eggs; fruits and vegetables; fats and oils) consumed three or more times a day. One point was awarded for each food category for a maximum DDS of 6 points. A higher DDS reflects a more diverse diet [32]. To assess caregiver perceptions and concerns about child weight, caregivers were asked what they thought about, whether they worried about, or if they had ever attempted or were currently attempting to alter their children’s weights. Beliefs about overweight/obesity were evaluated on a 5-point Likert scale with possible responses from “strongly agree” to “strongly disagree.” Lastly, caregivers were presented with a series of silhouette charts and asked to select (1) the image that they believed corresponded with their children’s current figure and (2) the image that matched their ideal figure for their children. Seven silhouettes, which were adapted from a previous study, were provided for both boys and girls ranging from very thin to very fat [33]. 

### 2.3. Anthropometric Measurements

Body mass index (BMI) was calculated by dividing body weight (kg) by height-squared (m^2^). Cutoff values for normal, overweight, and obese determinations were evaluated using age- and sex-specific BMI cutoffs developed for Chinese children and adolescents [34]. Parental BMI was calculated from self-reported height and weight collected from the questionnaire to determine whether there was an association between parental and child weight status. Child height and weight measurements were taken from the most recent physical examinations conducted by the schools, all of which occurred within half a year of the study. Participant age was calculated for the time at which weight and height measurements were collected.

### 2.4. Statistical Analysis

Chi-squared tests and *t*-tests were used to examine differences in dietary behaviors and caregiver perceptions by weight status. Multivariable logistic regression was used to identify factors associated with caregiver underestimation of child weight and evaluate whether inaccurate caregiver perception of weight was associated with specific dietary habits and caregiver attitudes. Participants with missing data were excluded from the final analysis. Data entry was completed in EpiData 3.1 (EpiData Association, Odense, Denmark) and statistical analyses were conducted using IBM SPSS Statistics v25 (IBM Corp., Armonk, NY, USA). Formal IRB approval was obtained from Yale University and Central South University prior to the start of the study.

## 3. Results

### 3.1. Participant Characteristics

A total of 424 questionnaires were collected. After excluding participants with missing data and those who were not between 2 and 6 years of age, the effective sample size was 364. The completion rate of the questionnaire was 85.8%. No significant differences were observed between included and excluded children for weight status, age, sex, or primary caregiver. Baseline characteristics by weight status are shown in Table 1. The mean age of the final sample (*n* = 364) was 4.4 ± 0.8 years. There was an equal distribution of males and females in the sample (181 male, 183 female). Over half of participants (53.3%) had a monthly household income of over 11,000 RMB. Estimates suggest that monthly incomes between 5000 and 19,000 RMB are considered middle class in China [35]. Mothers were the most common primary caregiver (68.1%), followed by grandparents (23.4%), and fathers (7.1%). About half (52.5%) of primary caregivers had above a college level education, while 28.6% of caregivers’ highest level of education completed was senior secondary school or below. The prevalence of overweight and obesity for the entire sample was 12.9% and 4.9%, respectively. Males had a slightly higher prevalence than females (19.3% vs. 16.4%), although this difference was not statistically significant. Both mothers and fathers of overweight/obese children had significantly higher BMIs than those of children with normal weight statuses (*p* = 0.029, *p* = 0.008 respectively).

### 3.2. Dietary Behaviors and Caregiver Attitudes

Differences in dietary behaviors by child weight status were assessed using bivariate analysis and are shown in Table 2. 

Overweight/obese participants spent significantly less time on meals with 15.4% of overweight/obese children spending less than 15 min on meals compared to 4.7% of children with a normal BMI (*p* = 0.013). Furthermore, compared to children with a normal weight status, significantly fewer overweight/obese children walked or played while eating (43.1% vs. 60.5%, *p* = 0.010), held food in their mouths (29.2% vs. 44.1%, *p* = 0.027), and had a poor appetite (52.3% vs. 85.6%, *p* < 0.001). There was no significant difference between normal-weight and overweight/obese children for being a picky eater, having fixed meal times or amounts, or having a dietary diversity score greater than 4. Among caregivers of overweight/obese children, 52.3% said that they worried about their children’s weight, 55.4% said that they would not wish to change their children’s weights, and 41.5% wanted their children to be thinner (Table 3). 

These rates were significantly different from those reported by caregivers of normal-weight children, with fewer caregivers worrying about child weight and wishing for their children to maintain or lose weight (*p* = 0.035, *p* < 0.001 respectively). When caregivers of overweight/obese children were asked what they thought about their children’s weight statuses, 58.5% responded normal and 38.5% said overweight/obese. Again, this differed significantly from perceptions of caregivers with normal-weight children, 56.9% of whom responded normal weight and 1.7% of whom responded overweight (*p* < 0.001). Overall, 61.6% of caregivers with overweight/obese children underestimated their children’s weight status. Significantly more caregivers of overweight/obese children than those with normal-weight children selected a heavier figure when asked to select a body shape that matched their children’s (16.9% vs. 1.0%, *p* < 0.001). However, there was no significant difference in the ideal body shape caregivers selected for their children, with about 80% in both categories selecting a moderate figure. There was also no significant difference in caregiver beliefs about overweight/obesity and its impact on health and future well-being. Significantly more caregivers of overweight/obese children adopted measures to control their children’s weights, such as exercise (*p* = 0.038), eating less meat (*p* < 0.001), eating less rice (*p* = 0.006), and reducing overall food intake (*p* < 0.001). More caregivers of normal-weight children also attempted to increase their children’s food intake as a means of altering their weights (33.8% vs. 7.7%, *p* < 0.001).

### 3.3. Factors Associated with Inaccurate Caregiver Perception of Child Weight Status

As shown in Table 4, a multivariable logistic regression model adjusted for child BMI indicated that caregivers were significantly more likely to underestimate the weights of sons than daughters (OR: 1.768; 95% CI: 1.129, 2.770, *p* = 0.013). Those with a monthly family income between 7000 and 11,000 RMB were also more likely to underestimate their children’s weight compared to those with an income greater than 11,000 RMB (OR: 1.697; 95% CI: 1.042, 2.762, *p* = 0.033). However, there was no significant association for age, maternal BMI, primary caregiver, or caregiver education level.

### 3.4. Associations between Caregiver Misperceptions, Dietary Behaviors, and Caregiver Attitudes

Multivariable logistic regression models adjusted for age, BMI, income, and education level were conducted to evaluate factors associated with caregiver underestimation of child weight (Table 5). Dietary behaviors, caregiver attitudes, and caregiver efforts to alter child weight were independently assessed for caregivers of overweight/obese children who underestimated child weight. We found that children with caregivers that underestimated weight were significantly more likely to have a poor appetite (OR: 5.883; 95% CI: 1.461, 23.693, *p* = 0.013). For caregiver attitudes, those who underestimated weight were significantly less likely to worry about their children’s weights (OR: 0.023, 95% CI: 0.003, 0.193, *p* = 0.001) and more likely to wish their children would maintain their current weight or gain weight (OR: 47.065; 95% CI: 6.374, 347.489, *p* < 0.001). Lastly, in terms of measures employed to alter weight, caregivers who underestimated weight were significantly less likely to restrict children’s diets by reducing meat consumption (OR: 0.136; 95% CI: 0.026, 0.711, *p* = 0.018) or overall food intake (OR: 0.189; 95% CI: 0.041, 0.874, *p* = 0.033) and more likely to refrain entirely from employing measures to change their children’s weights (OR: 13.061; 95% CI: 1.138, 0149.959, *p* = 0.039).

## 4. Discussion

We report a high prevalence (61.6%) of caregiver underestimation of overweight/obesity among urban, Chinese preschoolers. Caregivers with male children and those from lower-middle income households were significantly more likely to underestimate their children’s overweight/obese weights. Furthermore, caregivers who underestimated weight were more likely to have children with poor appetites, less likely to worry about child weight status, and less likely to adopt dietary restrictions for their children. These findings suggest that accurate classification of child weight is an important factor in shaping the dietary behaviors for young children and may be a vital component of future interventions to manage pediatric overweight/obesity. 

The high prevalence of overweight/obesity among young, Chinese children (17.8%) reported in this investigation was consistent with previous findings in Chinese populations [1,2,3,4]. Similar to findings in China, the US, and industrialized European countries, we also found a large proportion of caregivers with overweight/obese children underestimated their children’s weight status [23,24,27,28,29]. However, to our knowledge, no studies have previously been conducted in China evaluating caregiver perceptions among children as young as 2-years old. Given the high prevalence of overweight among preschool-aged children reported in this study and others, early interventions should be prioritized for the management of pediatric overweight/obesity [1,2]. Interventions that focus on parenting and the early feeding environment have been suggested to be effective, which highlights the need to understand the role that caregiver perceptions play in shaping child weight during infancy and early childhood [16].

Caregivers in our study were more likely to inaccurately classify the weights of male children than female children, which was consistent with previous findings among Chinese adolescents [27]. The difference in ability to discriminate between the weights of male and female children may be attributable to gender norms. Whereas slender body types tend to be more favorable for girls, overweight boys may be perceived as strong [36]. As a result, overweight daughters may elicit greater attention and scrutiny than sons. We also found that caregivers from households earning 7000 to 11,000 RMB per month were significantly more likely to underestimate child weight compared to those from households earning more than 11,000 RMB. However, there was no significant association for households earning less than 7000 RMB per month. This study is the first to our knowledge to report an association between lower-middle income households and caregiver underestimation of child weight status in China. Health knowledge and education among caregivers may be a potential explanation for our findings. In qualitative studies with low-income families in the US, mothers preferred to describe their overweight children as tall or big boned and believed that their children would grow out of being overweight [37,38]. However, further work is necessary to understand the association between household income and caregiver perceptions.

Inability to accurately identify children’s overweight statuses is problematic given that substantial evidence suggests that perceived level of concern is critical for motivating health-related behavioral changes [21,22]. Furthermore, studies demonstrate that parents play a pivotal role in the successful management of childhood obesity [17,18,19,20]. We found that caregivers of overweight/obese children who underestimated their children’s weights were less likely to worry about weight and more likely to want their child to maintain their current weight or gain weight. These results suggest that caregiver misperceptions of children’s overweight statuses may pose significant barriers to positive change. 

A previous study conducted in the US reported that parental misperception of child weight was associated with unhealthy dietary behaviors [39]. Specifically, we found that overweight/obese children with caregivers who underestimated their weights were more likely to have a poor appetite. Although there is evidence that supports an underlying genetic basis for appetite, parents have also been shown to play a critical role in the development of child eating behaviors and appetite [15,18]. It may be speculated that caregivers who fail to recognize their children’s overweight/obese weight statuses will also be less attentive to their dietary behaviors. It is also possible that caregivers of children with poor appetites are more likely to perceive them as undernourished, leading caregivers to underestimate their weight. The development of appetite in Chinese children is poorly understood and further work is required to fully elucidate the relationship between child appetite and caregiver perceptions. 

Caregivers in our study who incorrectly classified child weight were less likely to implement dieting measures for their children, such as eating less meat and reducing overall dietary intake. This positive association between caregiver accuracy and dietary restriction is similar to findings from a US study, which reported that parents who correctly identified their children as overweight were more likely to encourage dieting [40]. Additionally, we found that 12.5% of caregivers who underestimated their children’s overweight/obese status said that they attempted to increase food intake for their children (compared to 0% of caregivers who accurately classified child weight). Encouraging increased food consumption and restricting dietary intake have both been associated with long-term weight gain [39,40,41]. The casual relationships between feeding practices and child weight gain are not well defined and may depend on more nuanced factors, such as parenting style. In our previous study, we found that parental BMI values were positively correlated with child BMI [42]. Nevertheless, our findings identify well-intentioned, but potentially detrimental dietary practices employed by caregivers seeking to control their children’s weights. 

Several limitations should be noted when interpreting our results. First, our investigation employed a cross-sectional design in a medium sized city in southern China. As a result, we were unable to report causality in our results and our findings may not be representative of the entire country. Second, we used self-reported data from caregivers, which were subject to social-desirability and other forms of response bias. Parental height and weight were also based on self-reported values, which may have been biased or inaccurate. Third, height and weight measurements of children came from physical examinations conducted by each school. This data may have been subject to measurement errors and inter-observer agreement was not assessed. Fourth, although our questionnaire was adapted from previously validated instruments and tested among Chinese populations, it was not validated in this particular study population [29,32]. Lastly, 14.2% of participants were excluded from the final analysis, primarily due to missing data. There were no significant differences, however, in weight status, age, sex, or primary caregiver identity between the included and excluded populations. 

## 5. Conclusions

In summary, the large proportion of caregiver misperceptions about child weight status reported in this study is concerning, especially due to the high prevalence of pediatric overweight/obesity in China. Given that caregiver underestimation of weight was significantly associated with low concern regarding overweight/obesity, interventions that encourage accurate caregiver identification of child weight may be critical to motivating healthy behavioral changes. Furthermore, our results reveal potential biases among Chinese caregivers with male children and those from lower-middle income households that warrant further exploration. Future research should also include longitudinal or case-control studies that seek to characterize the relationship between caregiver attitudes and eating habits in Chinese children.

## Figures and Tables

**Table 1:** Demographic and household characteristics of participants by weight status.

	Normal (*n* = 299)		Overweight/Obese (*n* = 65)		Total (*n* = 364)		*p*-Value
	*n*	*%*	*n*	*%*	*n*	*%*	
**Sex**							0.463
Male	146	(48.8)	35	(53.8)	181	(49.7)	
Female	153	(51.2)	30	(46.2)	183	(50.3)	
**Age**							0.800
<4	101	(33.8)	25	(38.5)	126	(34.6)	
4–5	120	(40.1)	27	(41.5)	147	(40.4)	
5–6	66	(22.1)	11	(16.9)	77	(21.2)	
6–7	12	(4.0)	2	(3.1)	14	(3.8)	
**Only child**							0.976
Yes	188	(62.9)	41	(63.1)	229	(62.9)	
No	111	(37.1)	24	(36.9)	135	(37.1)	
**Monthly income**							0.204
<7000 RMB	35	(11.7)	3	(4.6)	38	(10.4)	
7000–11,000 RMB	105	(35.1)	27	(41.5)	132	(36.2)	
>11,000 RMB	159	(53.2)	35	(53.8)	194	(53.3)	
**Primary caregiver**							0.937
Mother	202	(67.6)	46	(70.8)	248	(68.1)	
Father	22	(7.4)	4	(6.2)	26	(7.1)	
Grandparent	70	(23.4)	15	(23.1)	85	(23.4)	
Other	5	(1.7)	0	(0.0)	5	(1.4)	
**Primary caregiver’** **s education**							0.204
≤Senior secondary school	86	(28.8)	18	(27.7)	104	(28.6)	
College	60	(20.1)	9	(13.8)	69	(19.0)	
>College	153	(51.2)	38	(58.5)	191	(52.5)	
**Birth weight (kg)**	3.410 ± 0.839		3.338 ± 0.502		3.398 ± 0.789		0.506
**Mother’s BMI**	21.029 ± 2.319		21.867 ± 2.835		21.178 ± 2.436		0.029 *
**Father’s BMI**	23.816 ± 2.776		24.776 ± 2.543		23.988 ± 2.757		0.008 *

Differences were analyzed using Chi-squared tests; * *p* < 0.05, statistically significant difference.

**Table 2:** Differences in dietary behaviors between normal-weight and overweight/obese children.

	Normal (*n* = 299)		Overweight/Obese (*n* = 65)		Total (*n* = 364)		*p*-Value
	*n*	*%*	*n*	*%*	*n*	*%*	
**Fixed meal times**							0.615
Often/daily	255	(85.3)	57	(87.7)	312	(85.7)	
Never/rarely	44	(14.7)	8	(12.3)	52	(14.3)	
**Fixed meal amount**							0.406
Often/daily	210	(70.2)	49	(75.4)	259	(71.2)	
Never/rarely	89	(29.8)	16	(24.6)	105	(28.8)	
**Time spent on meals**							0.013 *
<15 min	14	(4.7)	10	(15.4)	24	(6.6)	
15–30 min	177	(59.2)	33	(50.8)	210	(57.7)	
>30 min	108	(36.1)	22	(33.8)	130	(35.7)	
**Needs to be spoon-fed**							0.068
Yes	242	(80.9)	46	(70.8)	288	(79.1)	
No	57	(19.1)	19	(29.2)	76	(20.9)	
**Walks/plays while eating**							0.010 *
Yes	181	(60.5)	28	(43.1)	209	(57.4)	
No	118	(39.5)	37	(56.9)	155	(42.6)	
**Holds food in mouth**							0.027 *
Yes	132	(44.1)	19	(29.2)	151	(41.5)	
No	167	(55.9)	46	(70.8)	213	(58.5)	
**Poor appetite**							<0.001 *
Yes	256	(85.6)	34	(52.3)	290	(79.7)	
No	43	(14.4)	31	(47.7)	74	(20.3)	
**Picky eater**							0.527
Yes	249	(83.3)	52	(80.0)	301	(82.7)	
No	50	(16.7)	13	(20.0)	63	(17.3)	
**DDS (≥** **5 of 6)**	217	(72.6%)	45	(69.2%)	262	(72.0%)	0.586

Differences were analyzed using Chi-squared tests; * *p* < 0.05, statistically significant difference; DDS = Dietary Diversity Score.

**Table 3:** Differences in caregiver attitudes, beliefs, and efforts pertaining to child weight between normal-weight and overweight/obese children.

Caption	Normal (*n* = 299)		Overweight/Obese (*n* = 65)		Total (*n* = 364)		*p*-Value
	*n*	*%*	*n*	*%*	*n*	*%*	
**Do you worry about your child’s weight?**							0.035 *
Yes	114	(38.1)	34	(52.3)	148	(40.7)	
No	185	(61.9)	31	(47.7)	216	(59.3)	
**What do you think about your child’s weight?**							<0.001 *
Underweight	124	(41.5)	2	(3.1)	126	(34.6)	
Normal	170	(56.9)	38	(58.5)	208	(57.1)	
Overweight/obese	5	(1.7)	25	(38.5)	30	(8.2)	
**What do you wish about your child’s weight?**							<0.001 *
Thinner	8	(2.7)	27	(41.5)	35	(9.6)	
No change	148	(49.5)	36	(55.4)	184	(50.5)	
Fatter	143	(47.8)	2	(3.1)	145	(39.8)	
**Which body shape matches your child’s?**							<0.001 *
Thinner figure (E–G)	124	(41.5)	9	(13.8)	133	(36.5)	
Moderate figure (D)	172	(57.5)	45	(69.2)	217	(59.6)	
Heavier figure (A–C)	3	(1.0)	11	(16.9)	14	(3.8)	
**Which is the ideal body shape for your child?**							0.890
Thinner figure (E–G)	49	(16.4)	12	(18.5)	61	(16.8)	
Moderate figure (D)	244	(81.6)	52	(80.0)	296	(81.3)	
Heavier figure (A–C)	6	(2.0)	1	(1.5)	7	(1.9)	
**Agree or strongly agree**							
Overweight children are more likely to be overweight/obese in adulthood	223	(74.6)	45	(69.2)	268	(73.6)	0.375
Overweight/obese children are less healthy	234	(78.3)	49	(75.4)	283	(77.7)	0.613
Being overweight/obese impacts interpersonal relationships	177	(59.2)	41	(63.1)	218	(59.9)	0.563
**Measures employed to alter child’s weight**							
Exercise	170	(56.9)	46	(70.8)	216	(59.3)	0.038 *
Eat less meat	16	(5.4)	13	(20.0)	29	(8.0)	<0.001 *
Eat less rice	5	(1.7)	6	(9.2)	11	(3.0)	0.006 *
Reduce food intake	10	(3.3)	21	(32.3)	31	(8.5)	<0.001 *
Increase food intake	101	(33.8)	5	(7.7)	106	(29.1)	<0.001 *
None of these	72	(24.1)	12	(18.5)	84	(23.1)	0.330

Differences were analyzed using Chi-squared tests; * *p* < 0.05, statistically significant difference.

**Table 4:** Odds ratios for caregiver underestimation of child weight status.

	Adjusted OR (95% CI)		*p*-Value
**Age**	0.958	(0.730, 1.259)	0.759
**Sex**			
Male	1.768	(1.129, 2.770)	0.013 *
Female			Reference
**Mother’s BMI Monthly income**	0.926	(0.841, 1.020)	0.118
<7000 RMB	1.491	(0.709, 3.135)	0.292
7000–11,000 RMB	1.697	(1.042, 2.762)	0.033 *
>11,000 RMB			Reference
**Primary caregiver**			
Mother	1.173	(0.558, 2.466)	0.673
Father	1..266	(0.415, 3.860)	0.679
Grandparent			Reference
**Primary caregiver education**			
≤Secondary school	1.518	(0.719, 3.202)	0.274
College	0.657	(0.352, 1.225)	0.186
>College			Reference

* *p* < 0.05, statistically significant difference; adjusted for student body mass index (BMI).

**Table 5:** Associations between caregiver perceptions of child weight and dietary habits for overweight/obese children.

Caption	Underestimated (*n* = 40) ^a^		
	OR (95% CI)		*p*-Value
**Dietary behaviors**			
Fixed meal times	0.352	(0.028, 4.369)	0.417
Fixed meal amounts	0.257	(0.041, 1.624)	0.149
Needs to be spoon-fed	0.230	(0.037, 1.406)	0.112
Walks/plays while eating	0.250	(0.057, 1.088)	0.065
Holds food in mouth	1.093	(0.473, 2.534)	0.904
Has a poor appetite	5.883	(1.461, 23.693)	0.013 *
Picky eater	1.764	(0.377, 8.243)	0.471
DDS (≥5 of 6)	0.377	(0.028, 4.995)	0.459
**Caregiver attitudes**			
Worries about child’s weight	0.023	(0.003, 0.193)	0.001 *
Wishes child would maintain or gain weight	47.065	(6.374, 347.489)	<0.001 *
Moderate ideal body shape	1.842	(0.365, 9.282)	0.459
**Measures employed to alter child weight**			
Exercise	0.317	(0.072, 1.406)	0.131
Eat less meat	0.136	(0.026, 0.711)	0.018 *
Eat less rice	0.247	(0.025, 2.447)	0.232
Reduce food intake	0.189	(0.041, 0.874)	0.033 *
None of these	13.061	(1.138, 149.959)	0.039 *

^a^ Caregivers who accurately identified child weight were used as the reference group; * *p* < 0.05, statistically significant difference; adjusted for age, body mass index (BMI), income, caregiver education.

## Abbreviations

The following abbreviations are used in this manuscript:

DDS	Dietary Diversity Score
BMI	Body Mass Index
RMB	Ren Min Bi
